# Gender-Specific Relationships of Lifestyle and Physical Activity with Body Composition Among Medical Students

**DOI:** 10.3390/jcm15218753

**Published:** 2026-11-09

**Authors:** Jovana Maricic, Djurdja Nikic, Aleksa Ostojic, Matija Obrenovic, Filip Nikolic, Nikola Topalovic, Milica Zekovic, Rada Jeremic, Dejan Nesic, Sanja Mazic, Marina Djelic Blagojevic

**Affiliations:** Laboratory for Sport Medicine and Exercise Therapy, Institute of Medical Physiology, Faculty of Medicine, University of Belgrade, 11000 Belgrade, Serbia; dj.nikic@gmail.com (D.N.); ostojicaleksa.aki@gmail.com (A.O.); matijaobrenovic1@gmail.com (M.O.); topalovic21@yahoo.com (N.T.); rdjeremic@yahoo.com (R.J.);

**Keywords:** body composition parameters, medical students, physical activity

## Abstract

**Background/Objectives**: During their university education, medical students may develop unhealthy lifestyle behaviors during university education, including reduced physical activity, increased sedentary behavior, and inadequate dietary habits, which may affect their body composition. This study aimed to identify demographic, lifestyle, and physical activity predictors of body composition among medical students. **Methods**: This cross-sectional study included 176 undergraduate medical students from the Faculty of Medicine, University of Belgrade. Demographic characteristics, lifestyle habits, and physical activity were assessed using the questionnaire. Body composition was measured using the InBody 370S multifrequency bioelectrical impedance analyzer. Student’s *t*-test and chi-square test were used to assess between group differences. Univariate and multivariate linear regression analyses were performed separately for males and females. **Results**: Most students had normal BMI (76.1%), while 15.3% were overweight and 2.8% obese. Men had higher mean BMI (24.2 ± 3.4 vs. 21.5 ± 2.5 kg/m^2^) and lower body fat percentage (16.6 ± 5.3% vs. 27.0 ± 6.3%) than women (*p* < 0.001). In males, sport participation was associated with total body water (*p* = 0.008) and fat-free mass (*p* = 0.016), while vigorous physical activity predicted protein mass (*p* = 0.002). In females, sport participation was associated with total body water (*p* = 0.029), fat-free mass (*p* = 0.030), protein (*p* = 0.020), and skeletal muscle mass (*p* = 0.021). **Conclusions**: Sport participation was related to both higher total body water and fat-free mass in both males and females, and, was additionally related to higher protein and skeletal muscle mass in females.

## 1. Introduction

The student population comprises young adults whose health behaviour, including good dietary habits, adequate sleep, and sufficient physical activity, is essential for their mental, cognitive, and physical development and well-being [1,2]. Medical students represent a key population for the promotion of healthy lifestyles, as they will become future healthcare providers and will be involved in educating patients about disease prevention and healthy lifestyle practices. To date, only a limited number of studies addressing nutritional knowledge and habits, levels of physical activity, and different health-related parameters in this student population, have been conducted, with various methodological limitations.

At the beginning of their studies, students generally report having healthy lifestyle habits and making efforts to maintain an appropriate lifestyle in terms of diet, physical activity, and sleep, in accordance with contemporary recommendations. However, research has shown that, throughout their university education, medical students frequently experience reduced levels of physical activity, inadequate, irregular, and unhealthy dietary patterns, insufficient sleep, and increased sedentary behavior as a result of academic, professional, and personal challenges. Once students enter university, irregular meals, fast food consumption, insufficient physical activity, and inadequate sleep may negatively affect their health and contribute to overweight and obesity [3,4,5], and it has been estimated that 20–40% of the global student population is obese [1,6,7].

Although the body mass index (BMI) is widely used to assess weight status, it does not distinguish between fat mass and fat-free mass or provide information about body fat distribution [8]. Body composition assessment therefore offers a more detailed characterization of an individual’s physical status by differentiating between adipose and lean tissues and quantifying parameters such as percentage body fat, skeletal muscle mass, and total body water. This information is particularly relevant for young adults, in whom individuals with similar BMI values may have substantially different body composition profiles. Therefore, the present study focused on body composition to provide a more comprehensive assessment of the associations between lifestyle factors, physical activity, and body composition in medical students. Body composition parameters, including the distribution of muscle, adipose, and bone tissue, as well as the amounts of water, protein, minerals, and other components, are most commonly assessed using bioelectrical impedance analysis with a device known as a body composition analyzer. This method is based on the transmission of electrical impulses through different types of tissue via electrodes, with the impulses encountering varying levels of tissue resistance and reactance [9]. Maintaining an adequate and balanced body composition is important for preserving good health. Excessive body fat is associated with an increased risk of various cardiovascular diseases, diabetes, and hypertension [10]. Visceral fat (intra-abdominal fat) refers to adipose tissue surrounding the organs within the abdominal cavity, and elevated levels are associated with the development of various diseases [11]. In contrast, a greater amount of muscle mass is associated with higher energy expenditure and basal metabolic rate and may contribute to greater stability and balance, which is important in preventing injuries such as falls and fractures [12].

Numerous factors may influence body composition parameters among students, including substantial academic demands, rigorous schedules, insufficient sleep, increased sedentary behavior, and reduced engagement in physical activity due to limited free time and motivation, among others reasons [13,14]. Furthermore, limited evidence is available regarding differences in body composition between male and female university students and the factors associated with or underlying these differences. In the very few studies conducted on this topic, these sex differences appear to indicate that males and females respond differently in terms of diet and body composition at the beginning of their university studies and that males demonstrated greater overall adverse changes [15]. Therefore, future research should focus on mitigating these adverse changes particularly during times of stress through the development of effective sex-specific interventions that mitigate adverse body composition changes. The World Health Organization (WHO) defines physical activity as any bodily movement produced by skeletal muscles that requires energy expenditure. This encompasses all movement, including movements performed during leisure time or as part of an individual’s occupation with moderate and vigorous-intensity physical activity being shown to improve health [16].

Although previous studies have examined physical activity and body composition among medical students, the factors associated with body composition in this population remain incompletely characterized. In particular, to avoid relying exclusively on conventional anthropometric indicators or physical activity measures, further research is needed on the associations between demographic, lifestyle, and physical activity-related factors and multiple body composition parameters, rather than relying exclusively on conventional anthropometric indicators or physical activity measures. Moreover, as body composition differs between males and females, examining these associations separately by sex may help to identify factors associated with body composition within each group and provide a more detailed understanding of the relationships under investigation. Therefore, in the present study our aim was to identify the demographic, lifestyle, and physical activity-related factors associated with body composition in medical students through sex-stratified analyses.

## 2. Materials and Methods

This is cross-sectional study took place at the Laboratory for Sport Medicine and Exercise Therapy, Institute of Medical Physiology, Faculty of Medicine, University of Belgrade, Belgrade, Serbia, during February of 2025. The study sample consisted of undergraduate medical students enrolled in all six years of study at the Faculty of Medicine, University of Belgrade (N = 176). Inclusion criteria were an active undergraduate student status, and a signed written informed consent. Exclusion criteria: comprised pregnancy or lactation, use of medications affecting body composition or fluid balance (e.g., corticosteroids), and rejection of study participation.

Prior to questionnaire completion and body composition assessment, all participants received written information explaining the study objectives, procedures, and the voluntary nature of participation. Written informed consent was obtained from all participants before enrolment. The study was approved by the Ethics Committee of the Faculty of Medicine, University of Belgrade (No. 27/I-13; 27 January 2025).

Data on demographic characteristics and lifestyle habits were collected using a questionnaire, previously developed at the Institute of Epidemiology, Faculty of Medicine, University of Belgrade, which has been used in various published studies, with certain modifications. Participants provided information regarding their usual daily lifestyle habits, including sedentary behavior, and physical activity levels, in the previous year. Completion of the questionnaire required approximately 15 min.

The questionnaire also included questions regarding the presence of chronic diseases and frequency of alcohol consumption (beer, wine, and spirits). Smoking status was defined according to the definition provided by the Centers for Disease Control and Prevention [17]. Students who reported smoking at least 100 cigarettes in their lifetime and who, at the time of the survey, smoked every day or some days were classified as current smokers. Students who reported smoking at least 100 cigarettes in their lifetime and who, at the time of the survey, did not smoke at all were defined as former smoker, and non-smokers were those individuals who did not classify themselves in either of these two groups.

Participants also reported the frequency of engaging in moderate-intensity physical activity lasting at least 30 min per session during a typical week. According to the WHO, moderate-intensity physical activity generally corresponds to approximately 3.0–5.9 metabolic equivalents (METs) with examples including brisk walking, dancing, gardening, cycling, swimming, and household activities [18]. Vigorous-intensity physical activity requires a large amount of effort and results in rapid breathing and a substantial increase in heart rate. Vigorous-intensity physical activity generally corresponding to 6.0 METs or more. Examples include running, fast cycling, fast swimming, and other activities requiring substantial physical effort [18]. Physical activity is broader than sport, with sport and exercise representing specific forms of physical activity [19]. The questionnaire assessed physical activity across only planned exercise, with the intensity of physical activity self-reported by participants based on MET values. More specifically, respondents were asked how often they practice physical activity of a certain category.

Sports participation, recreational physical activity, and vigorous physical activity were assessed using self-reported questionnaire items. With these variables were treated as separate indicators of physical activity-related behaviors; however, some overlap between them is possible. The questionnaire assessed the frequency of the reported activities but did not record session duration. Consequently, total physical activity volume could not be estimated, and the data were insufficient to determine whether participants met the recommended weekly physical activity levels. Physical activity was assessed using a questionnaire covering sports participation, recreational physical activity, type of sport, and self-reported physical activity during adolescence. Sports participation frequency was categorized as never, monthly, weekly, or daily. Recreational physical activity, described in the questionnaire as activities such as 20 min of exercise or brisk walking, was categorized as rarely, twice per week, or daily. Participants who reported practicing a sport could specify the type of sport from a predefined list or indicate another sport. Physical activity was assessed separately for light physical activity, described as activity without sweating or breathlessness, and strenuous physical activity, described as activity accompanied by sweating and increased breathing. For both categories, frequency was classified as none, less than once per week, 1–2 times per week, or three or more times per week. These intensity descriptions were based on participants’ self-reports and were not defined using metabolic equivalent (MET) values.

Sedentary behavior was defined according to the Sedentary Behavior Research Network consensus definition as waking behavior characterized by an energy expenditure of ≤1.5 METs while sitting, reclining, or lying down [20]. In the present study, sedentary time was assessed using a self-reported questionnaire item asking participants how many hours per day they spent sitting. Response options were: (a) up to 3 h, (b) up to 5 h, (c) up to 8 h, and (d) more than 8 h per day. Sitting time was considered in the context of students’ daily activities, including studying, attending classes, and leisure activities such as watching television or using electronic devices. The measure reflected participants’ self-reported sitting time and did not distinguish between specific sedentary activities. The questionnaire did not specify particular sitting activities, distinguish between weekdays and weekends, or ask participants to estimate a weekly average. Accordingly, the item was interpreted as a self-reported estimate of daily sitting time. The original response categories did not define mutually exclusive boundaries for the first three options, which limits the precision with which sitting time can be categorized.

Before body composition assessment, body height (cm) was measured using a medical stadiometer. Body composition was assessed using the InBody 370S analyzer (InBody Co., Seoul, Republic of Korea), a multifrequency bioelectrical impedance analysis (BIA) device. Measurements were performed according to the manufacturer’s recommendations, with participants standing barefoot on the platform while holding the hand electrodes. The device performs 15 impedance measurements across 5 body segments (both arms, trunk, and both legs) at 3 different frequencies, providing a comprehensive assessment of body composition, including body weight, total body water, protein mass, mineral content, skeletal muscle mass, body fat mass, percent body fat, body mass index (BMI), basal metabolic rate, segmental lean and fat mass, and other body composition parameters. Participants were classified according to the sex-specific reference ranges provided by the manufacturer [21]. The reference range for PBF was 10–20% for male participants and 18–28% for female participants. Values below the respective reference range were classified as “Under,” values within the reference range as “Normal,” and values above the reference range as “Over,” in accordance with the categories displayed in the device’s Obesity Analysis section (InBody, InBody 370S User Manual/Product Catalog). Measurements were performed in the morning, before the participants’ first meal of the day. Other pre-measurement conditions, including bladder emptying, recent fluid and alcohol intake, and recent physical activity, were not systematically documented. Therefore, compliance with all manufacturer-recommended preparation procedures could not be verified.

### Statistical Analysis

Descriptive statistics were used to summarize the study variables. Differences between groups for continuous variables were assessed using Student’s *t*-test, whereas categorical variables were compared using the chi-square (χ^2^) test. Lavene’s test of equality of variances was used to check the homogeneity of variance. If Levene’s test was significant (*p* < 0.05), indicating unequal variances, Welch’s *t*-test was applied instead.

To assess the different predictors of body composition parametar values, univariate and multivariate linear regression analyses were performed with the measured values of selected factors as dependent variables (BMI, percent of body fat, total body water, fat-free mass, minerals, proteins, skeletal muscle mass), and demographic and lifestyle characteristics and physical activity as independent variables. All variables significant in the univariate analysis at the 0.05 level were included in the multivariate models. Given the importance of sex as a potential confounding factor, analyses were conducted separately for male and female students. We assessed multicollinearity among the candidate predictors using the variance inflation factor (VIF).

The statistical significance threshold was set at *p* < 0.05. All analyses were performed using IBM SPSS Statistics for Windows, Version 21.0 (IBM Corp., NY, USA).

## 3. Results

Students from all six years of study at the FMUB participated in this study, and their demographic characteristics are presented in Table 1. Of the total of 176 participants, 64 (36.4%) were male, and 112 (63.6%) were female. The mean age of the participants was 23.0 ± 2.1 years, with no statistically significant difference between sexes (*p* = 0.384). The majority of participants (63.6%) lived in their own home, and regarding living arrangements, 48.9% of them lived with their parents, with no statistically significant differences between sexes (Table 1).

The distribution of participants according to smoking habits and frequency of alcohol consumption is shown in Table 2. Most participants were non-smokers (87.2%), while 11.4% were current smokers and 1.2% were former smokers. A statistically significant difference between sexes was observed in terms of smoking status (*p* = 0.018), with the prevalence of smoking being higher among females (16.1%) compared to males (3.2%). Regarding alcohol consumption, no statistically significant difference was observed between sexes (*p* = 0.365) (Table 2).

Table 3 shows the distribution of participants according to sports participation, recreational activities, and sedentary behavior. Regarding sports participation, there were no statistically significant difference between sexes in terms of frequency of sports participation (*p* = 0.152) and recreational activities (*p* = 0.226). Regarding the intensity of physical activity, no statistically significant difference between sexes was observed (*p* = 0.374). Moderate-intensity physical activity was performed three or more times per week by 67.6% of participants, once to twice per week by 26.1%, and less than once per week by 4.0%, while 2.3% reported never engaging in moderate-intensity physical activity, with no statistically significant difference between sexes (*p* = 0.540). Additionally, no statistically significant difference between sexes was observed for daily sedentary time (*p* = 0.102) (Table 3).

The participants body composition parameter distributions are presented in Table 4. The mean height of the participants was 173.5 ± 9.9 cm, with a mean height of 183.1 ± 6.1 cm among men and 167.9 ± 7.0 cm among women (*p* = 0.001). The mean body weight was 67.8 ± 14.6 kg, with a mean weight of 80.9 ± 13.8 kg among men and 60.4 ± 8.7 kg among women (*p* = 0.001). The mean total body water was 38.7 ± 10.2 L, protein mass 10.4 ± 2.7 kg, mineral mass 3.7 ± 0.9 kg, body fat mass 15.5 ± 6.0 kg, fat-free mass 52.6 ± 13.3 kg, and skeletal muscle mass 29.2 ± 8.1 kg, with statistically significant differences between sexes were observed for all analyzed body composition parameters (*p* = 0.001) (Table 4).

Table 5 presents the distribution of participants according to categories of BMI and BFP, along with their mean values. The mean BMI for the entire sample was 22.5 ± 3.1 kg/m^2^, with a mean BMI of 24.2 ± 3.4 kg/m^2^ among men and 21.5 ± 2.5 kg/m^2^ among women (*p* = 0.001). The distribution of BMI categories differed significantly between sexes (*p* = 0.001). The mean BFP was 23.2 ± 7.8% for the entire sample, 16.6 ± 5.3% among men and 27.0 ± 6.3% among women (*p* = 0.001). Normal BFP values were observed in 69.3% of participants, including 79.7% of men and 63.4% of women. The difference in the distribution of BFP categories between sexes was not statistically significant (*p* = 0.062) (Table 5).

To determine which factors were statistically significantly related to body composition parameter values, we performed several univariate linear regression analyses, separately for male and female students, with these parametar values as the dependent variable, and different demographic and lifestyle factors as independent variables (Table 6 and Table 7), separately for male and female students. All variables which were statistically significant (*p* < 0.05) in univariate analyses, entered into the multivariate linear regression models. Our analysis showed that in male students higher sedentary time during the day (β = 1.126, 95%CI 0.098–2.153, *p* = 0.032; N-sample size = 64, n-number of variables in the model = 4) was the most significant contributor to BMI. Participation in sport was statistically significantly associated with the total body water (β = 3.290, 95%CI 0.980–5.671, *p* = 0.008; N = 64, *n* = 4) and fat-free mass (β = 3.591, 95%CI 0.698–6.485, *p* = 0.011; N = 64, *n* = 2), while participation in recreational activities (β = 0.278, 95%CI 0.067–0.489, *p* = 0.011; N = 64, *n* = 3) and vigorous physical activity (β = 0.185, 95%CI 0.021–0.349, *p* = 0.027; N = 64, *n* = 3) were associated with minerals levels. Participation in recreational activities (β = 2.892, 95%CI 1.117–4.667, *p* = 0.011; N = 63, *n* = 3) and vigorous physical activity (β = 1.626, 95%CI 0.232–3.021, *p* = 0.027; N = 64, *n* = 3) were also associated with skeletal muscle mass, while participation in sports (β = 0.770, 95%CI 0.212–1.329, *p* = 0.002; N = 63, *n* = 4) was also a predictor of protein mass in the body composition. Among female students, age (β = 0.312, 95%CI 0.122–0.502, *p* = 0.002; N = 112, *n* = 2) and smoking (β = −1.161, 95%CI −2.264–−0.058, *p* = 0.039; N = 112, *n* = 2) were the most significant contributor to BMI. Conversely, smoking (β = −3.771, 95%CI −6.547–−0.968, *p* = 0.009; N = 112, *n* = 2) and infrequent participation in sport (β = −2.537, 95%CI −4.317–−0.758, *p* = 0.006; N = 112, *n* = 2) were significant negative predictors of body fat percentage. Total body water (β = 0.785, 95%CI 0.084–1.486, *p* = 0.029; N = 112, *n* = 2), fat-free mass (β = 1.073, 95%CI 0.106–2.039, *p* = 0.030; N = 112, *n* = 2), protein level (β = 0.227, 95%CI 0.036–0.419, *p* = 0.020; N = 112, *n* = 2) and skeletal muscle mass (β = 0.676, 95%CI 0.105–1.248, *p* = 0.021; N = 112, *n* = 2) were significantly determined by sport participation.

In almost all models we did not detect multicollinearity, i.e., the VIF was about 1, which meant that the predictor variable was completely independent of the other variables.

## 4. Discussion

The relationship between body composition and physical activity in medical university students is complex and multifactorial, and our study confirms the existence of multiple relationships between body composition elements and physical activity in this group. The few studies of this type, conducted for this research group, have mainly been cross-sectional studies, in which considerable heterogeneity can be observed in both the number and type of parameters assessed. This represents an important research gap, highlighting the need for standardized reporting of body composition parameters. Given the different objectives of individual studies, their substantial methodological differences, and other circumstances influencing the selection of variables, it is necessary to define a minimum set and type of parameters that should be reported as standardized measures of body composition. This would enable meaningful comparisons between different subpopulations and facilitate the identification of factors that distinguish them. Such factors, whether they act in a positive or negative direction, could provide a basis for the developing preventive strategies [22].

Given the statistically significant differences obtained in our study between male and female students in terms of body weight and height, total body water, and protein, mineral, body fat, fat-free, and skeletal muscle masses, we decided to analyze the factors influencing body composition separately for the male and female populations. Additionally, all variables which were statistically significant in our univariate analysis, were entered into the multivariate linear regression models.

Our analysis showed that in male students higher sedentary time during the day (*p* = 0.032) was the most significant contributor to BMI; participation in sport was statistically significantly associated with total body water (*p* = 0.008) and fat-free mass (*p* = 0.016); participation in recreational activities (*p* = 0.016) and vigorous physical activity (*p* = 0.027) were associated with mineral levels and skeletal muscle mass (*p* = 0.002, *p* = 0.023), and vigorous physical activity (*p* = 0.002) was a predictor of protein mass in body composition. However, these results should be interpreted with caution, as the relationship between the sample size and the number of variables in multivariate linear regression analysis directly determines the stability, accuracy, and validity of the statistical model. In our study, this particularly applies to the male subgroup, where the sample size barely meets the mathematical minimum, and where stable coefficients and adequate statistical power require significantly larger samples (e.g., 50 subjects per variable).

Jaremkow et al. investigated body composition elements with the strongest correlations with physical activity in 75 young students from medical faculties [9] and found that the amount of moderate physical activity performed by sudents correlated with BMI, fat-free mass and bone mass. The authors explained these relationships by stating that vigorous and moderate physical activity increases fat-free mass, particularly that of bones and muscles. Having greater muscle mass makes the body more efficient at using energy, which leads to a higher metabolism, while moderate physical activity caused an increase in body mass and consequently BMI. However, this increase was not caused by an increase in fat tissue but rather by an increase in lean mass.

Female students were found to have significantly lower values for daily energy intake, muscle mass, visceral fat, and vitamin D levels [23], while male participants had significantly greater body weight and height, body mass index, fat-free mass, protein mass, mineral mass, total body water, and vital capacity, as well as lower body fat [24]. It is important to note that Zhang et al. demonstrated a positive correlation between fat mass and the vital capacity index among male students with a BMI < 18.5. In contrast to their findings, the study found no correlation between fat mass and pulmonary function in men [25].

In our study, among males, the relationship between sedentary time and BMI may reflect the potential contribution of prolonged sitting to lower daily energy expenditure. Medical students may spend considerable time studying, attending lectures, and using computers, potentially increasing sedentary time even among those who participate in sport. Research in university populations has demonstrated that sedentary behavior and physical activity can coexist and that their relationships with BMI and other anthropometric indicators may be complex [26]. Although students may participate in regular sports and physical activity, prolonged sitting during the remainder of the day may still contribute to lower overall daily energy expenditure, which could partly explain the observed association between sedentary time and BMI [27]. On the other hand, regular sport and vigorous exercise may contribute to the development and maintenance of skeletal muscle and other fat-free tissue. As total body water is closely related to fat-free mass, associations between sport participation and these two parameters may partly reflect their underlying physiological relationship [28]. Nevertheless, the specific type, duration, and frequency of sport, as well as dietary protein and energy intake, may influence these relationships. Since these mechanisms were not necessarily measured directly in the present study, they should be interpreted as possible explanations rather than established causal pathways.

In our study, it was demonstrated that, in female students, age (*p* = 0.002) was the most significant contributor to BMI, while smoking (*p* = 0.009) and infrequent participation in recreational activities (*p* = 0.006) were significant negative predictors of body fat percentage. In addition, total body water (*p* = 0.029), fat-free mass (*p* = 0.030), protein level (*p* = 0.020) and skeletal muscle mass (*p* = 0.021) were significantly determined by sport participation. In the female subgroup, the results of the linear regression are significantly more reliable because the number of predictors per model is slightly smaller, and the sample size is almost twice as large as in the male subgroup. Among female students, the associations between sport participation and total body water, fat-free mass, protein mass, and skeletal muscle mass suggest that sport participation is related to several interrelated components of lean tissue. This finding is broadly consistent with research among Italian university students, which found that associations between physical activity and body composition differed by sex. Zaccagni et al. reported that the most physically active males had a lower fat mass, whereas the most physically active females had greater fat-free mass [29]. Although the study population and methods differed from those of our investigation, these results provide a useful comparison and support the importance of evaluating body-composition components separately rather than relying exclusively on BMI. The association between age and BMI in females may reflect age-related differences in body weight, physical activity, dietary behavior, or body composition within the age range represented in the sample. Smoking was negatively associated with body fat percentage among females. Although nicotine exposure may influence appetite and energy expenditure, this finding should not be interpreted as evidence that smoking is beneficial for body composition or weight management [30]. Smoking may be associated with other behavioral and socioeconomic factors, and the observed relationship could reflect residual confounding, differences in lifestyle patterns, or characteristics of the sample [31]. The association between smoking and body composition observed in the present study should be interpreted cautiously. Falbová et al. (2023) reported higher fat mass, percentage body fat, and visceral fat area among regular smokers than among nonsmokers in young adults [32]. The difference between their findings and the inverse association observed among women in our study may reflect differences in smoking classification, smoking frequency, sample characteristics, or other lifestyle factors. Residual confounding by dietary habits, physical activity, and alcohol consumption may also have contributed to the observed association. Given the cross-sectional design and the self-reported nature of smoking data in the present study, no causal interpretation can be made.

The differences between the male and female models may also reflect differences in the variability and distribution of lifestyle exposures, the relatively small number of male participants compared with female participants, and correlations between body composition outcomes.

Our findings regarding physical activity-related differences in fat-free mass, skeletal muscle mass, protein mass, and total body water can be interpreted in the context of previous research in young adults. Falbová et al. (2024) reported associations between physical activity and several bioimpedance-derived body composition parameters, including fat-free mass, skeletal muscle mass, and total body water [33]. However, their study also examined differences related to the COVID-19 pandemic and infection status, which limits its direct comparability with the present study [34]. These findings nevertheless support the relevance of considering physical activity when interpreting bioimpedance-derived body composition estimates in young adults. The limitations of BMI as an indicator of body composition should also be considered. Falbová et al. (2025), in a study of young adults aged 18–30 years, reported that individuals with normal-weight obesity had higher visceral fat area and lower lean body mass in specific body segments despite having a normal BMI [35]. This supports the use of direct body composition assessment alongside BMI, as BMI does not distinguish fat mass from lean mass or account for differences in fat distribution. Nevertheless, the prevalence and classification of normal-weight obesity depend on the body fat percentage thresholds applied and the characteristics of the study population. Medical university students are important targets for promoting of healthy lifestyles in the adult population, due to their knowledge related to health issues and therefore, and opportunities to adopt of healthy practices. Besides that, medical university students are future physicians, and their habits and behaviors are most likely to become the norm [36]. Therefore, the findings obtained in our study may have significance for the creation of targeted strategies for the primary and secondary prevention of various chronic diseases and the protection of students health, and can also be used to define sex-specific and personalized preventive interventions.

Our study has several limitations. Our study design was cross-sectional, and data were collected from students at a single university based on convenience sampling, which may limit the generalizability of the results. Prospective cohort studies are a better study design option due to the greater reliability of the results, minimized selection bias and the ability to assess the effect of a single exposure on the occurrence of multiple health conditions. Additionally, a larger sample size would be beneficial, especially important to minimizing the risk of overfitting and unstable estimates. Furthermore, an a priori sample size calculation for the multivariate linear regression analyses was not performed. The generalizability of our findings may be limited by the study population, which consisted of medical students from a single institution in Belgrade, Serbia. Lifestyle behaviors, physical activity patterns, dietary habits, and body composition may differ across cultural, socioeconomic, and educational contextsand our findings may not be directly generalizable to medical students from other institutions or countries, or to university students in general. An additional limitation is that physical activity was assessed using self-reported frequency categories without recording session duration or objectively measuring activity intensity. Therefore, sport participation, recreational activity, and vigorous physical activity may overlap. Additionally, the validity and reliability of the questionnaire items were not formally established, and the broad response categories may have resulted in exposure misclassification. Although body composition measurements were performed in the morning before the participants’ first meal, other preparation conditions were not systematically controlled or recorded. In particular, the timing of bladder emptying, recent fluid and alcohol intake, and recent physical activity were not documented. These factors may have contributed to variability in bioelectrical impedance measurements and the resulting estimates of body composition, particularly total body water. Therefore, associations involving hydration-related parameters should be interpreted with caution. Additionally, the total physical activity volume could not be calculated, and compliance with recommended physical activity levels could not be established. Finally, our regression analyses were conducted separately for male and female students, and formal interaction tests between sex and the respective predictors were not performed. Furthermore, differences in subgroup sizes and small numbers in some response categories may have reduced the statistical power and the precision of regression estimates. Residual confounding by unmeasured lifestyle and demographic factors cannot be excluded. Finally, the examination of multiple body composition outcomes and lifestyle predictors increases the possibility of chance findings. These limitations should be considered when interpreting the observed associations, particularly those identified in sex-stratified analyses. Future studies involving larger and more diverse student populations are warranted to determine whether these findings can be replicated across different settings.

## 5. Conclusions

In this study, statistically significant differences in the levels of body composition parameters were detected between male and female medical students. Additionally, in both male and female medical students, increased participation in sport was significantly determined by improvements in hydration and fat-free mass. Furthermore, in females, protein level and skeletal muscle mass were significantly determined by sport participation. In males, participation in recreational activities was associated with mineral levels and skeletal muscle mass, while vigorous physical activity was also a predictor of protein mass in the body composition. Further studies are warranted to confirm the sex-specific relationships observed in our study.

## Figures and Tables

**Table 1:** Demographic characteristics of the study participants.

Variable	Values			*p*
	Both Sexes (N = 176)	Men (*n* = 64)	Women (*n* = 112)	
Age (years) *	23.0 ± 2.1	22.8 ± 1.8	23.1 ± 2.3	0.384
Place of residence - Own home - Rented apartment - Student dormitory - Other	112 (63.6) 36 (20.5) 26 (14.8) 2 (1.1)	42 (65.6) 10 (15.6) 12 (18.8) 0 (0.0)	70 (62.5) 26 (23.2) 14 (12.5) 2 (1.8)	0.330
Living arrangements - Living alone - Living with parents - Sharing an apartment with a roommate - Living with relatives - Living with a partner	33 (18.8) 86 (48.9) 41 (23.3) 13 (7.4) 3 (1.6)	13 (20.3) 29 (45.3) 15 (23.4) 7 (10.9) 0 (0.0)	20 (17.9) 57 (50.9) 26 (23.2) 6 (5.4) 3 (2.7)	0.431

* mean ± standard deviation.

**Table 2:** Distribution of the participants according to smoking habits and frequency of alcohol consumption.

Variable	Values			*p*
	Both Sexes (N = 176)	Men (*n* = 64)	Women (*n* = 112)	
Smoking - Current smoker - Non-smoker - Former smoker	20 (11.4) 153 (87.2) 2 (1.2)	2 (3.2) 61 (96.8) 0 (0.0)	18 (16.1) 92 (82.1) 2 (1.8)	**0.018**
Alcohol - Does not consume - Once per month - Once per week - Twice per week - Four or more times per week	28 (16) 59 (33.7) 77 (44) 9 (5.1) 2 (1.0)	14 (22.2) 20 (31.7) 25 (39.7) 4 (6.4) 0 (0.0)	14 (12.5) 39 (34.8) 52 (46.4) 5 (4.5) 2 (1.8)	0.365

One missing response was identified within the male subgroup. Bold values denote statistical significance.

**Table 3:** Distribution of participants according to sports participation and recreational physical activities.

Variables	Values			*p*
	Both Sexes (N = 176)	Men (*n* = 64)	Women (*n* = 112)	
Sports participation - Never - Monthly - Weekly - Daily	25 (14.2) 24 (13.6) 89 (50.6) 38 (21.6)	6 (9.4) 12 (18.8) 29 (45.3) 17 (26.6)	19 (17.0) 12 (10.7) 60 (53.6) 21 (18.7)	0.152
Participation in recreational activities - Rarely - Twice per week - Daily	17 (9.7) 60 (34.1) 99 (56.2)	9 (14.1) 22 (34.4) 33 (51.5)	8 (7.1) 38 (33.9) 66 (59)	0.226
Vigorous physical activity - Never - Less than once per week - One to two times per week - Three or more times per week	13 (7.4) 25 (14.2) 56 (31.8) 82 (46.6)	5 (7.8) 8 (12.5) 16 (25) 35 (54.7)	8 (7.1) 17 (15.2) 40 (35.7) 47 (42.0)	0.374
Moderate physical activity - Never - Less than once per week - One to two times per week - Three or more times per week	4 (2.3) 7 (4.0) 46 (26.1) 119 (67.6)	1 (1.6) 3 (4.7) 13 (20.3) 47 (73.4)	3 (2.7) 4 (3.6) 33 (29.5) 72 (64.3)	0.540
Sedentary time during the day - Up to three hours - Four to five hours - Six to eight hours - More than eight hours	9 (5.1) 58 (33.1) 94 (53.7) 14 (8.1)	6 (9.4) 24 (37.5) 28 (43.8) 6 (9.3)	3 (2.7) 34 (30.6) 66 (59.5) 8 (7.2)	0.102

One missing response identified in the female subgroup for the variable ‘sedentary time during the day’.

**Table 4:** Distribution of participants according to body composition parameters.

Variable	Values			*p*
	Both Sexes (N = 176)	Men (*n* = 64)	Women (*n* = 112)	
Height (cm)	173.5 ± 9.9	183.1 ± 6.1	167.9 ± 7.0	***p* < 0.001**
Weight (kg)	67.8 ± 14.6	80.9 ± 13.8	60.4 ± 8.7	***p* < 0.001**
Total body water (L)	38.7 ± 10.2	50.0 ± 8.0	32.2 ± 3.6	***p* < 0.001**
Protein mass (kg)	10.4 ± 2.7	13.4 ± 2.0	8.6 ± 1.0	***p* < 0.001**
Mineral mass (kg)	3.7 ± 0.9	4.6 ± 0.7	3.2 ± 0.4	***p* < 0.001**
Body fat mass (kg)	15.5 ± 6.0	13.5 ± 6.1	16.7 ± 5.6	***p* = 0.001**
Fat-free mass (kg)	52.5 ± 13.3	67.4 ± 9.7	44.0 ± 5.0	***p* < 0.001**
Skeletal muscle mass (kg)	29.2 ± 8.1	38.4 ± 5.8	24.0 ± 3.0	***p* < 0.001**

Bold values denote statistical significance.

**Table 5:** Distribution of participants according to body mass index (BMI) and body fat percentage.

Variable	Values			*p*
	Both Sexes (N = 176)	Men (*n* = 64)	Women (*n* = 112)	
Body mass index—BMI (kg/m^2^)	22.5 ± 3.1	24.2 ± 3.4	21.5 ± 2.5	***p*** ** < 0.001**
- Underweight	10 (5.7%)	2 (3.1%)	8 (7.1%)	**0.001**
- Normal weight	134 (76.1%)	39 (60.9%)	95 (84.8%)	
- Overweight	27 (15.3%)	19 (29.7%)	8 (7.1%)	
- Obesity	5 (2.8%)	4 (6.2%)	1 (1%)	
Body fat percentage—BFP (%)	23.2 ± 7.8	16.6 ± 5.3	27.0 ± 6.3	***p*** ** < 0.001**
- Normal BFP	122 (69.3%)	51 (79.7%)	71 (63.4%)	0.062
- Mildly elevated BFP	24 (13.6%)	7 (10.9%)	17 (15.2%)	
- High BFP	30 (17.0%)	6 (9.4%)	24 (21.4%)	

Bold values denote statistical significance.

**Table 6:** Predictors of body composition parameters in male students.

Variables	Univariate Analysis			Multivariate Analysis				
	Unstandardized Beta Coefficient	95% CI	*p*	Unstandardized Beta Coefficient	95% CI	*p*	Model Sample Size	Adjusted R^2^
	BMI							
Age	−0.033	−0.525–0.460	0.895				64	0.157
Place of residence	0.956	−0.115–2.026	0.076					
Living arrangements	0.388	−0.568–1.345	0.420					
Smoking	4.313	−0.588–9.214	0.083					
Alcohol consumption	0.348	−0.644–1.340	0.486					
Presence of comorbidities	0.309	−1.652–2.271	0.754					
Sports participation - Never - Monthly - Weekly - Daily	1.304	0.404–2.205	**0.005**	0.679	−0.497–1.855	0.253		
Participation in recreational activities - Rarely - Twice per week - Daily	1.139	0.007–2.271	**0.049**	0.210	−1.100–1.519	0.750		
Sedentary time during the day - Up to three hours - Four to five hours - Six to eight hours - More than eight hours	1.211	0.158–2.265	**0.025**	1.126	0.098–2.153	**0.032**		
Moderate physical activity	0.347	−0.999–1.693	0.608					
Vigorous physical activity - Never - Less than once per week - One to two times per week - Three or more times per week	1.057	0.191–1.924	**0.018**	0.771	−0.175–1.718	0.108		
	**Percent of body fat**							
Age	0.721	−0.020–1.462	0.056					
Place of residence	1.474	−0.184–3.133	0.081					
Living arrangements	−0.029	−1.519–1.461	0.969					
Smoking	0.054	−7.734–7.842	0.989					
Alcohol consumption	1.098	−0.420–2.616	0.153					
Presence of comorbidities	−1.694	−4.705–1.317	0.265					
Sports participation	−1.049	−2.512–0.414	0.157					
Participation in recreational activities	−1.096	−2.885–0.693	0.225					
Sedentary time during the day	1.575	−0.078–3.228	0.062					
Moderate physical activity	0.240	−1.850–2.329	0.819					
Vigorous physical activity	0.095	−1.310–1.500	0.893					
	**Total body water**							
Age	−0.426	−1.572–0.720	0.460				64	0.249
Place of residence	1.587	−0.947–4.121	0.215					
Living arrangements	−0.054	−2.302–2.195	0.962					
Smoking	10.321	−1.120–21.762	0.076					
Alcohol consumption	0.922	−1.394–3.238	0.429					
Presence of comorbidities	2.319	−2.231–6.870	0.312					
Sports participation - Never - Monthly - Weekly - Daily	4.284	2.322–6.246	**<0.001**	3.290	0.908–5.671	**0.008**		
Participation in recreational activities - Rarely - Twice per week - Daily	4.410	1.919–6.902	**0.001**	2.094	−0.781–4.969	0.150		
Sedentary time during the day	0.090	−2.476–2.665	0.944					
Moderate physical activity	2.358	−0.738–5.454	0.133					
Vigorous physical activity - Never - Less than once per week - One to two times per week - Three or more times per week	3.056	1.083–5.029	**0.003**	1.482	−0.581–3.545	0.156		
	**Body fat mass**							
Age	0.783	−0.071–1.638	0.072					
Place of residence	1.663	−0.247–3.573	0.087					
Living arrangements	0.213	−1.926–1.500	0.805					
Smoking	2.457	−6.480–11.393	0.585					
Alcohol consumption	0.525	−1.246–2.296	0.556					
Presence of comorbidities	−1.890	−5.356–1.575	0.280					
Sports participation - Never - Monthly - Weekly - Daily	0.616	−1.088–2.319	0.473					
Participation in recreational activities	0.682	−1.394–2.757	0.514					
Sedentary time during the day	2.356	0.494–4.219	**0.014**					
Moderate physical activity	0.742	−1.655–3.139	0.538					
Vigorous physical activity	1.394	−0.184–2.971	0.082					
	**Fat-free mass**							
Age	−0.610	−1.990–0.770	0.380				64	0.241
Place of residence	1.473	−1.599–4.544	0.342					
Living arrangements	0.811	−1.893–3.515	0.551					
Smoking	13.352	−0.395–27.098	0.057					
Alcohol consumption	−0.091	−2.899–2.717	0.949					
Presence of comorbidities	2.162	−3.345–7.669	0.436					
Sports participation - Never - Monthly - Weekly - Daily	4.956	2.561–7.352	**<0.001**	3.591	0.698–6.485	**0.016**		
Participation in recreational activities	5.403	2.408–8.399	**0.001**					
Sedentary time during the day	1.791	−1.270–4.851	0.247					
Moderate physical activity	2.829	−0.906–6.564	0.135					
Vigorous physical activity - Never - Less than once per week - One to two times per week - Three or more times per week	3.693	1.315–6.072	**0.003**	1.916	−0.586–4.417	0.105		
	**Minerals**							
Age	−0.022	−0.118–0.74	0.647				64	0.189
Place of residence	0.103	−0.109–0.314	0.337					
Living arrangements	0.046	−0.141–0.233	0.624					
Smoking	0.785	−0.171–1.741	0.106					
Alcohol consumption	0.013	−0.181–0.206	0.897					
Presence of comorbidities	0.104	−0.277–0.484	0.589					
Sports participation - Never - Monthly - Weekly - Daily	0.290	0.119–0.462	**0.001**	0.119	−0.103–0.341	0.289		
Participation in recreational activities - Rarely - Twice per week - Daily	0.343	0.133–0.553	**0.002**	0.278	0.067–0.489	**0.011**		
Sedentary time during the day	0.124	−0.087–0.335	0.245					
Moderate physical activity	0.207	−0.050–0.464	0.112					
Vigorous physical activity - Never - Less than once per week - One to two times per week - Three or more times per week	0.244	0.079–0.409	**0.004**	0.185	0.021–0.349	**0.027**		
	**Proteins**							
Age	−0.126	−0.404–0.152	0.367				63	0.033
Place of residence	0.305	−0.314–0.923	0.329					
Living arrangements	0.167	−0.377–0.712	0.541					
Smoking	2.815	0.056–5.577	**0.046**	1.406	−1.173–3.985	0.280		
Alcohol consumption	−0.010	−0.576–0.556	0.973					
Presence of comorbidities	0.466	−0.643–1.575	0.404					
Sports participation - Never - Monthly - Weekly - Daily	1.031	0.552–1.509	**<0.001**	0.770	0.212–1.329	0.008		
Participation in recreational activities - Rarely - Twice per week - Daily	1.119	0.519–1.719	**<0.001**	0.587	−0.124–1.299	0.104		
Sedentary time during the day	0.362	−0.255–0.979	0.245					
Moderate physical activity	0.557	−0.197–1.310	0.145					
Vigorous physical activity - Never - Less than once per week - One to two times per week - Three or more times per week	0.754	0.275–1.232	**0.003**	0.375	−0.146–0.895	0.155		
	**Skeletal muscle mass**							
Age	−0.383	−1.203–0.437	0.354				63	0.256
Place of residence	0.683	−1.148–2.515	0.459					
Living arrangements	0.387	−1.223–1.996	0.633					
Smoking	8.288	0.136–16.439	**0.046**	3.726	−3.801–11.253	0.326		
Alcohol consumption	−0.100	−1.769–1.570	0.905					
Presence of comorbidities	1.238	−2.037–4.513	0.453					
Sports participation - Never - Monthly - Weekly - Daily	2.960	1.537–4.382	**<0.001**	1.260	−0.634–3.155	0.188		
Participation in recreational activities - Rarely - Twice per week - Daily	3.440	1.686–5.193	**<0.001**	2.892	1.117–4.667	**0.002**		
Sedentary time during the day	1.172	−0.644–2.987	0.202					
Moderate physical activity	1.571	−0.655–3.797	0.163					
Vigorous physical activity - Never - Less than once per week - One to two times per week - Three or more times per week	2.193	0.778–3.607	**0.003**	1.626	0.232–3.021	**0.023**		

CI-confidence interval. Bold values denote statistical significance.

**Table 7:** Predictors of body composition parameters in female students.

Variables	Univariate Analysis			Multivariate Analysis				
	Unstandardized Beta Coefficient	95% CI	*p*	Unstandardized Beta Coefficient	95% CI	*p*	Model Sample Size	Adjusted R^2^
	BMI							
Age	0.319	0.126–0.512	**0.001**	0.312	0.122–0.502	**0.002**	112	0.124
Place of residence	0.149	−0.449–0.747	0.622					
Living arrangements	−0.242	−0.710–0.226	0.308					
Smoking	−1.224	−2.373–−0.075	**0.037**	−1.161	−2.264–−0.058	**0.039**		
Alcohol consumption	0.062	−0.497–0.621	0.826					
Presence of comorbidities	−0.704	−1.627–0.218	0.133					
Sports participation	0.329	−0.157–0.815	0.183					
Participation in recreational activities	−0.383	−1.124–0.357	0.308					
Sedentary time during the day	0.068	−0.671–0.808	0.855					
Moderate physical activity	0.190	−0.483–0.862	0.577					
Vigorous physical activity	0.099	−0.409–0.607	0.700					
	**Percent of body fat**							
Age	0.368	−0.139–0.876	0.153				112	0.125
Place of residence	0.420	−1.097–1.938	0.584					
Living arrangements	−0.089	−1.282–1.104	0.883					
Smoking	−4.385	−7.241–−1.529	**0.003**	−3.771	−6.547–−0.968	**0.009**		
Alcohol consumption	0.293	−1.124–1.709	0.683					
Presence of comorbidities	−1.037	−3.385–1.320	0.385					
Sports participation	−0.882	−2.113–0.349	0.159					
Participation in recreational activities - Rarely - Twice per week - Daily	−2.905	−4.711–−1.099	**0.002**	−2.537	−4.317–−0.758	**0.006**		
Sedentary time during the day	0.508	−1.356–2.372	0.590					
Moderate physical activity	−0.422	−2.128–1.285	0.625					
Vigorous physical activity	−0.994	−2.269–0.282	0.125					
	**Total body water**							
Age	0.125	−0.167–0.417	0.399				112	0.070
Place of residence	−0.067	−0.936–0.802	0.878					
Living arrangements	−0.160	−0.842–0.522	0.643					
Smoking	1.241	−0.443–2.926	0.147					
Alcohol consumption	0.417	−0.390–1.224	0.308					
Presence of comorbidities	−0.137	−1.490–1.215	0.841					
Sports participation - Never - Monthly - Weekly - Daily	0.929	0.240–1.617	**0.009**	0.785	0.084–1.486	**0.029**		
Participation in recreational activities	0.696	−0.375–1.768	0.201					
Sedentary time during the day	−0.341	−1.411–0.729	0.529					
Moderate physical activity	0.470	−0.503–1.443	0.340					
Vigorous physical activity - Never - Less than once per week - One to two times per week - Three or more times per week	0.832	0.111–1.552	**0.024**	0.643	−0.084–1.371	0.083		
	**Body fat mass**							
Age	0.515	0.069–0.960	**0.024**	0.542	0.122–0.962	**0.012**	112	0.150
Place of residence	0.155	−1.198–1.507	0.821					
Living arrangements	−0.217	−1.279–0.845	0.686					
Smoking	−3.633	−6.190–−1.076	**0.006**	−2.970	−3.855–−0.724	**0.005**		
Alcohol consumption	0.615	−0.641–1.872	0.334					
Presence of comorbidities	−1.033	−3.130–1.064	0.331					
Sports participation	−0.481	−1.583–0.621	0.389					
Participation in recreational activities - Rarely - Twice per week - Daily	−2.433	−4.050–−0.817	**0.004**	−2.586	−4.165–−1.060	**0.002**		
Sedentary time during the day	0.518	−1.146–2.182	0.539					
Moderate physical activity	−0.193	−1.713–1.328	0.802					
Vigorous physical activity	−0.505	−1.649–0.639	0.383					
	**Fat-free mass**							
Age	0.169	−0.233–0.571	0.405				112	0.241
Place of residence	−0.105	−1.301–1.092	0.863					
Living arrangements	−0.222	−1.161–0.717	0.640					
Smoking	1.686	−0.633–4.006	0.153					
Alcohol consumption	0.580	−0.531–1.690	0.303					
Presence of comorbidities	−0.187	−2.050–1.675	0.842					
Sports participation - Never - Monthly - Weekly - Daily	1.267	0.319–2.216	**0.009**	1.073	0.106–2.039	**0.030**		
Participation in recreational activities	0.946	−0.529–2.422	0.206					
Sedentary time during the day	−0.427	−1.901–1.047	0.567					
Moderate physical activity	0.647	−0.693–1.986	0.341					
Vigorous physical activity - Never - Less than once per week - One to two times per week - Three or more times per week	1.130	0.138–2.123	**0.026**	2.882	−0.641–6.406	0.107		
	**Minerals**							
Age	0.011	−0.019–0.040	0.472					
Place of residence	−0.004	−0.092–0.084	0.935					
Living arrangements	−0.012	−0.081–0.058	0.741					
Smoking	0.066	−0.106–0.238	0.451					
Alcohol consumption	0.060	−0.021–0.142	0.145					
Presence of comorbidities	−0.014	−0.151–0.123	0.836					
Sports participation	0.070	−0.001–0.140	0.054					
Participation in recreational activities	0.033	−0.077–0.142	0.556					
Sedentary time during the day	−0.012	−0.120–0.097	0.833					
Moderate physical activity	0.041	−0.058–0.140	0.410					
Vigorous physical activity	0.063	−0.011–0.136	0.096					
	**Proteins**							
Age	0.036	−0.044–0.116	0.374				112	0.073
Place of residence	−0.024	−0.262–0.214	0.842					
Living arrangements	−0.045	−0.231–0.142	0.636					
Smoking	0.372	−0.088–0.832	0.112					
Alcohol consumption	0.094	−0.127–0.315	0.399					
Presence of comorbidities	−0.051	−0.421–0.319	0.785					
Sports participation - Never - Monthly - Weekly - Daily	0.265	0.077–0.453	**0.006**	0.227	0.036–0.419	**0.020**		
Participation in recreational activities	0.208	−0.085–0.501	0.162					
Sedentary time during the day	−0.079	−0.371–0.214	0.596					
Moderate physical activity	0.118	−0.149–0.384	0.383					
Vigorous physical activity - Never - Less than once per week - One to two times per week - Three or more times per week	0.223	0.025–0.420	**0.027**	0.168	−0.031–0.367	0.097		
	**Skeletal muscle mass**							
Age	0.106	−0.132–0.345	0.379				112	0.075
Place of residence	−0.075	−0.785–0.635	0.834					
Living arrangements	−0.135	−0.692–0.422	0.632					
Smoking	1.094	−0.281–2.468	0.118					
Alcohol consumption	0.283	−0.378–0.943	0.398					
Presence of comorbidities	−0.175	−1.280–0.930	0.755					
Sports participation - Never - Monthly - Weekly - Daily	0.792	0.231–1.353	**0.006**	0.676	0.105–1.248	**0.021**		
Participation in recreational activities	0.620	−0.254–1.495	0.163					
Sedentary time during the day	−0.271	−1.146–0.604	0.540					
Moderate physical activity	0.361	−0.434–1.157	0.370					
Vigorous physical activity - Never - Less than once per week - One to two times per week - Three or more times per week	0.682	0.094–1.271	**0.023**	0.520	−0.073–0.113	0.085		

CI-confidence interval. Bold values denote statistical significance.

## Data Availability

The datasets generated during and/or analyzed during the current study are available from the corresponding author on reasonable request.
